# Clinical Observations on Continued Fever Occurring in This Climate

**Published:** 1892-11

**Authors:** Everard H. Richardson

**Affiliations:** Atlanta, Ga.


### CLINICAL OBSERVATIONS ON CONTINUED FEVER OCCURRING IN THIS CLIMATE.*

BY EVERARD H. RICHARDSON, M. D.
ATLANTA, GA.

Having only a few hours at my command in the preparation of this article, necessarily its scope will be circumscribed and its character superficial. In the brief thesis of the evening no attempt will be made to elaborate a critical essay upon the causation or pathology of continued fevers. But I shall confine myself to impressions and convictions made upon my mind concerning the character and treatment of that form of continued fever that has very often prevailed in this section of country during the past decade. I assume the position that all continued fevers occurring in this climate where we are able to exclude all other forms of fever, and which sulphate of quinine administered in large doses for three consecutive nights fails to abort or jugulate, are essentially cases of typhoid fever, and should

*Read before the Atlanta Society of Medicine.

be treated as such. I am aware that many will think this rule arbitrary and dogmatic, but I consider it approximately correct and a *good* working rule in practice. I do not believe in the existence of that form of fever described by some of our eminent book makers as "continued malarial fever," persisting after the proper administration of sulphate of quinine without some co-existing complication. Attempts at the description of continued fevers have been essayed since the days of Hippocrates. Autopsies made during the seventeenth century revealed the intestinal lesions of typhoid fever. It, however, was reserved for the nineteenth century to define the distinctive intestinal lesions of typhoid fever, and to differentiate it from other forms of fever. The name "typhoid" was given to this disease in the year 1829 by Louis. Up to this period typhoid and typhus fever were regarded as identical; the occurrence of the intestinal lesions of the former was considered an accidental complication of typhus fever. But Gerhard, of Philadelphia, a student of Louis, shortly after this demonstrated the difference between the two diseases. This knowledge, first established by Gerhard, was widely disseminated in America before it was recognized in Europe or Great Britain. But for the writer to enter into a minute, detailed description of the phenomena and history of a stereotyped case of typhoid fever would be a task void of interest or profit. The typical cases of typhoid fever with great prostration of the nervous system, lasting from four to six weeks, requires but little discrimination or medical acumen to recognize. The main object of this paper is to deal with the *atypical* or *anomalous* form of fever occurring in this section, and which I think should be denominated *atypical typhoid fever.* In recent years quite a number of these cases have come under my personal observation and from conversations upon this subject with a number of my professional *confrères* I am not alone in my views of the character of this type of fever. However, I confess my inability to change the views of those members of the profession who have the old impression of typhoid fever mirrored upon their mind by observations of the disease twenty or thirty years ago. In recent years readers of our medical journals have noticed the unanimity of opinion advanced with reference to the change in type of typhoid fever. The tympanites which formerly was always present, and by many regarded as pathognomonic of typhoid fever, coming on about the end of the first week, is not now by any means a constant accompaniment of the disease. On the contrary a retracted belly is often present. The premonitory symptoms are also modified in intensity and of shorter duration as a rule than the prodromic period of the average case of typhoid fever. The temperature in these cases is subject to the same rise in the evening and fall in the morning, as in the typical cases of typhoid fever. The evening temperature by the fourth day may reach 104° F. If the temperature should reach 104° F. earlier it points to a co-existing malarial element. The pain in the head is usually a prominent symptom ; and slight pain, with gurgling in the right iliac region, though not invariably present, is very often detected by the *tactus eruditus*. I have rarely observed the tendency to diarrhœa or the rose colored spots in this type of fever, both of which many authors consider characteristic of typical typhoid fever. But the most conspicuous symptom of regular typhoid fever that is absent is the profound prostration of the nervous system, as well as the shorter duration of the disease. Delirium is entirely absent. The patient in a number of instances visits the office of the physician with the story that he has not been well for a week or longer, does not feel able to attend to his business nor sufficiently ill to go to bed ; the most prominent symptoms are pain in the head, which is worse in the evening, moderate lassitude and loss of appetite. You take his temperature and find it perhaps 102° F. Say to this patient that he has an attack of fever and it is best for him to take his bed and submit to treatment and if he is an individual of strong will power and great energy he will be inclined to doubt your wisdom ; but if he has confidence in your honesty and judgment he follows your advice. You administer large doses of quinine for two or three days, which lowers his temperature somewhat and perhaps upsets his stomach, and perturbs his nervous system, but does not abridge or modify the course of the fever.

The clinical history of these cases shows that very many of them terminate in from ten to twelve days, leaving the patient with but little prostration. The evening temperature of this class of cases may never exceed 103° F. The pulse ranges from 96 to 116 beats per minute. I have the record of a case treated last year in Atlanta where the temperature was normal on the eleventh day. This patient insisted upon getting up, and remained clear of fever for several days, but relapsed, and when I again visited her found a temperature in the evening of 102° F. and a pulse of 108. In three days without an antipyretic this patient was again without fever and convalesced rapidly.

In June of the present year I treated Mr. C., aged 26 years, for this form of fever. He first visited me in my office on June 8. His temperature was only a little above 101° F.; pulse 96 per minute. Acting upon my advice he went to his home, and I visited him June 9, 10, 11 and 14. On the nights of 8th, 9th and 10th I gave him twenty grains of sulphate of quinine which had no effect in interrupting the progress of the fever. On the evening of the 14th for the first time he was free from fever, and remained so. The evening temperature in this case at no time ran higher than 103° F. Supposing that this gentleman had fever for three days before seen, in this event his fever terminated in ten days. He sat up in his room during the day, and complained only of pain in the head, loss of appetite and inability to sleep at night. Three weeks after his recovery his young wife, six months pregnant, was taken with a typical case of typhoid fever. During her attack she had two hemorrhages from her bowels. Shortly after the end of the second week she miscarried and one week later she died from exhaustion. The hemorrhages from the bowels occurred prior to the miscarriage and undoubtedly came from the bowels.

This case presented from its inception all the symptoms of typical typhoid fever; and I doubt not a *post mortem* examination would have shown the characteristic intestinal lesions incident to the *later stages* of the disease. Doubtless these two cases occurring in the same family and in the same house originated from the same cause, or possibly the wife was infected from the husband.

A few years ago it was my lot to treat a case of continued fever and remain by the bedside of the patient during the entire attack. It was in a locality where typhoid fever was prevailing at the time. Dr. J. B. S. Holmes, of Rome, Ga., saw the case several times and confirmed the diagnosis of typhoid fever. I am absolutely sure that in this case there was no fever after the thirteenth day. During the past twenty years it has been my fortune to make a number of *post mortem* examinations in cases dying from continued fever. In each case save one of continued fever terminating in death the examination revealed the intestinal lesions of typhoid fever.

An eminent modern medical writer declares that "as far back as our knowledge of the disease goes typhoid fever presents the same picture." No statement could hardly be further from the truth. I fear that some physicians make the same mistake that laymen fall into in concluding that a fever cannot be typhoid unless it is a well defined typical case lasting from four to six weeks. I think it to be true that enlarged experience convinces us that the clinical history of typhoid fever varies greatly. Not only so, but I am sure that the type from some unknown cause has been changed and modified. As a matter of fact the typical cases do occur now as formerly, but not so frequently in this climate as the modified form of the disease.

No disease differs in its history wider than the "ambulatory" or walking cases of typhoid fever and the severer types of the disease. And yet the cause of the disease doubtless is the same, the pathological changes differing only in the extent of the structural lesions produced either upon the nervous system or upon the walls of the intestinal tract. In the cases of typhoid fever where we never have fever after the tenth day, Caley gives a very specious explanation of the wide divergence in the duration of different cases of the disease. His theory is "that in typhoid we have two forms of fever to deal with—a primary, due to the infection of the system by the typhoid poison; and a secondary, caused by gangrene and the ulceration of the intestines and consequent septicæmia." This explanation is plausible and forcible and is in harmony with our present knowledge of the pathology of the disease. In the cases where convalescence is established in ten days we are warranted in concluding that the stages of ulceration and gangrene are never reached in the intestinal glands, never passing beyond the stages of congestion and hyperplasia, but happily terminating in resolution. Another type of the disease not mentioned is where death occurs in the first stage from the violence of the fever or from the overwhelming influence of the poison upon the nerve centers, though this is rare. The pathological lesions of typhoid fever, ulceration of the solitary and agminated glands of the ileum and extending to the solitary glands of the large intestines, it is reasonable to suppose are characteristic only of the *later stages* of the disease. I have already intimated my belief that the etiology of all forms of typhoid fever is the same. Notwithstanding the monopoly now enjoyed by bacteriologists and bacilli discoverers, and the association of typhoid microbes with the name of Klebs and Eberth, the credentials of the *bacillus typhosus* will not yet pass the muster roll. Nevertheless the fact remains beyond cavil or controversy that typhoid fever is produced by a specific poison, the habitat of which is filth, and that the poison, whatever it may be, in the vast majority of instances, enters the body through the alimentary canal, passing out with the evacuations from the affected subject, emphasizing the supreme importance of destroying this poison contained in the dejecta as early as possible, by the most reliable means at our command, in every *suspected* case of typhoid fever, and summoning every intelligent resource known to the most exalted and God-like branch of our profession, preventive medicine, for the laudable purpose of protecting the lives of both country and town by guarding well the water and food supplies of the people. In conclusion I cannot refrain from expressing my earnest belief in the opinion that in every case of continued fever, however mild its onset, it is the duty of the physician to treat it as though it might become the gravest. Who of us can predict whether a given case of fever will terminate in ten days or thirty days? Who can foresee a complication or anticipate a hemorrhage? I shall dismiss the subject of treatment in a very few words, alluding to it only in a general way. A large, clean, well-ventilated and quiet room is the first *desideratum;* an intelligent and faithful nurse should be procured.

The recumbent posture should be enjoined, the patient placed upon a liquid diet which should be regularly administered, and he should be made to sleep and everything done to promote general tranquillity. Unless perspiring the patient should be bathed twice daily in cold or tepid water, whichever is most pleasant to the subject. No other antipyretic should be used unless the temperature rises above 103° F. If sponging should fail to keep the temperature under 103° F. I consider phenacetine the safest antipyretic in our possession at the present time. The patient should be treated and not the disease; no perturbative measures should be used and every rational means instituted to foster the vital powers. In the beginning a mild purgative will not kill the patient, but after the first week the bowels should be controlled and should in this stage never be moved unless by an enema of warm water. After the first week should the pulse run beyond 120 beats per minute brandy should be given, as indications require, to prevent exhaustion. Opium in some form, barring idiosyncrasies, may in any stage be administered with propriety to relieve pain, control bowels or promote sleep, as well as to tranquillize the patient. I am no enthusiastic advocate or believer in the curative power of any special drug in the treatment of any form of typhoid fever. The treatment to be rational must be general. I know of no remedy that shortens the duration of any type of the disease. The complications and concomitants of typhoid fever and many of the symptoms of the disease, as well as the details of treatment, have not been reviewed for the reason that the writer deemed that such review would be of no interest to this body of medical gentlemen.

Before closing this article I wish to state that when the physician is certain that he has a case of continued fever to deal with, the administration of quinine in large doses is useless and not desirable on account of the sedation it produces and the tendency to disturb the nervous system. A *bacillus malariæ* in 1880 was discovered by Laveran in France. It is alleged that these parasitic organisms are always present in the blood of those suffering from any form of malarial poisoning. Osler avers "that the testimony is now unanimous in France, India, America, Italy and Germany that these bodies are always present in malarial fever. There is no evidence to show that they are ever present in any other disease." I have no personal experience in the examination of the blood of the subjects of malarial poisoning. But unfortunately we do not yet know the relationship of these organisms to malarial fever. If the dicta of Laveran and his disciples could be accepted as true it would represent a very great advance in our knowledge of the etiology of malarial fever, enabling us to differentiate this class of fever from all other forms by the examination of the blood. Laveran's method of diagnosing malaria if established would obviate the necessity for the administration of quinine in any form of fever except malarial, and if adding nothing to our knowledge of the treatment of malarial fever would entitle him to the lasting gratitude of mankind.

Whenever we are able with the microscope to diagnosticate with certainty typhoid and malarial fevers and to differentiate the one from the other by this simple method, though the knowledge may shed no new light on the treatment of these diseases, it will signalize a divine era in the history of medicine and will illume with effulgent and matchless splendor the darkest page in medicine—the etiology of disease.

